# Resources to Guide Exercise Specialists Managing Adults with Diabetes

**DOI:** 10.1186/s40798-019-0192-1

**Published:** 2019-06-03

**Authors:** Grant Turner, Scott Quigg, Peter Davoren, Renata Basile, Sybil A. McAuley, Jeff S. Coombes

**Affiliations:** 1 Chronic Disease and Post Acute Programs, Diagnostic, Emergency and Medical Services, Gold Coast Health, Queensland Health, Robina, Australia; 2 Metro North Hospital and Health Service, Community, Indigenous and Subacute Service, Diabetes Service, North Lakes Health Precinct, Queensland Health, North Lakes, Australia; 3 Division Medicine, Gold Coast Health, Queensland Health, Robina, Australia; 4 Diabetes Centre, Gold Coast University Hospital, Southport, Australia; 5 Department of Medicine, University of Melbourne, Melbourne, Australia; 6 Department of Endocrinology and Diabetes, St Vincent’s Hospital Melbourne, Melbourne, Australia; 7 School of Human Movement and Nutrition Sciences, University of Queensland, Brisbane, 4072 Australia

**Keywords:** Exercise, Glucose level, Hypoglycaemia, Type 1 diabetes, Type 2 diabetes

## Abstract

Exercise is an important element to optimize health and well-being, though navigating exercise safely can be challenging for exercise specialists working with people with diabetes. Measuring glucose levels before an exercise session assists in the determination of whether exercise is safe for a person with diabetes. A number of organizations have recently developed guidelines to provide exercise and diabetes recommendations based on glucose levels and other relevant factors. However, there are limited easy-to-use resources to assist exercise specialists to determine whether exercise should be started and continued by people with diabetes. The type of diabetes, pre-exercise glucose level, medications and their timing, recent food intake and general sense of wellness all warrant consideration when determining the approach to each exercise session. An expert group was convened to review the published literature and develop resources to guide exercise specialists in assessing the safety of an adult with diabetes starting exercise, and indications to cease exercise, based upon glucose levels and other factors. Contraindications to people with diabetes starting or continuing exercise are (1) glucose < 4.0 mmol/L; (2) glucose > 15.0 mmol/L with symptoms of weakness/tiredness, or with ketosis; (3) hypoglycaemic event within the previous 24 h that required assistance from another person to treat and (4) feeling unwell. To optimize diabetes and exercise safety, recommendations (stratified by pre-exercise glucose level) are provided regarding carbohydrate ingestion, glucose monitoring and medication adjustment.

**Electronic supplementary material:**

The online version of this article (10.1186/s40798-019-0192-1) contains supplementary material, which is available to authorized users.

## Key Points

- Guidelines are provided to optimize exercise for people with diabetes based on pre-exercise glucose level.
- Guidelines regarding glucose monitoring, carbohydrate ingestion, and medication adjustments are included
- Contraindications to exercise are provided.

## Introduction

Prescribing and delivering exercise to a person with diabetes requires an understanding of the interplay between the type of diabetes, the pre-exercise glucose level, medications and their timing, and recent food intake. The aim of this article is to present current recommendations as easy to use resources to assist exercise specialists to determine whether exercise should be started and continued by people with diabetes.

### Type 1 Diabetes and Exercise

Exercise is important for the health and well-being of people with type 1 diabetes. Cardiometabolic benefits include improvements in cardiorespiratory fitness, vascular function and lipid profile [1]. Physically active adults with type 1 diabetes have better blood pressure, a healthier BMI, lower requirements for insulin and less ketoacidosis than their physically inactive counter-parts [1, 2]. There also appears to be an association between physical activity and reduced cardiovascular disease and mortality for individuals with type 1 diabetes [3].

Current recommendations from the American Diabetes Association for people with type 1 diabetes are to accumulate at least 150 min/week of moderate-intensity aerobic and resistance exercise and to have no more than two consecutive days without physical activity [4]. However, more than 60% of people with diabetes undertake no structured exercise [2], and many individuals report that fear of exercise-induced hypoglycaemia and a lack of knowledge about the effects of exercise on glucose control are reasons they do not exercise [5].

Glucose homeostasis depends on the interaction between the nervous system, hormones (e.g., insulin, glucagon, catecholamines, and glucocorticoids), molecular regulators within skeletal muscle and the liver [6]. For people with type 1 diabetes, glucose control during exercise is challenging, as without the physiological response of insulin to exercise, deficiencies or exaggerations in other hormonal responses can occur. These responses may be difficult to predict, resulting in exercise causing either hypoglycaemia or hyperglycaemia for people with type 1 diabetes. The type of exercise further complicates the response with aerobic exercise tending to lower blood glucose and anaerobic exercise likely to increase glucose, making glycaemic control challenging [7]. During aerobic exercise, the lack of a physiological reduction in circulating insulin results in a lack of both physiological glucose production by the liver and increased skeletal muscle uptake of glucose. Together, these increase the risk of hypoglycaemia. During anaerobic exercise, a failure in circulating insulin levels to increase at the end of exercise and a rise in catecholamines increases glucose production by the liver. At the same time, glucose disposal into skeletal muscle is limited, resulting in hyperglycaemia. Knowledge of glucose levels and the direction of change expected during exercise may increase self-efficacy and confidence for exercise.

Exercise will generally increase the risk of hypoglycaemia for several hours following exercise for people with type 1 diabetes. Increased insulin sensitivity post-exercise appears to be biphasic, occurring immediately after exercise and then again 7–11 h later [8] and may last for up to 24 h [9]. It appears that, because the hormonal responses to exercise and hypoglycaemia are similar, they promote a cycle of repeated autonomic failure during both exercise- and insulin-induced hypoglycaemia [10]. The additional compounding problems with blunted autonomic nervous system and neuroendocrine and metabolic counter-regulatory responses are referred to as hypoglycaemia-associated autonomic failure (HAAF) [11]. Monitoring glucose levels and the direction of change following exercise can decrease post-exercise hypoglycaemia. This is especially important when there has been an increase in exercise (duration or intensity) or when a new exercise program is started.

### Type 2 Diabetes and Exercise

Exercise is medicine for people with type 2 diabetes. Exercise training improves glycaemic control, primarily by increasing glucose uptake into active muscles and inhibiting glucose production from the liver. Muscle glucose uptake is improved by insulin-dependent and insulin-independent pathways, and these benefits continue for several hours after exercise [12]. In addition to the effects on glycaemic control, exercise training also improves cardiovascular disease risk among people with type 2 diabetes by acting on hypercholesterolaemia, hypertension and obesity [13]. Furthermore, exercise leads to improved mental health and quality of life [13]. The extensive evidence regarding the health benefits of exercise has resulted in exercise training being incorporated into type 2 diabetes treatment guidelines throughout the world [14–17]. Current Australian guidelines recommend that people with type 2 diabetes or pre-diabetes accumulate a minimum of 210 min per week of moderate-intensity exercise or 125 min of vigorous-intensity exercise consisting of aerobic and resistance modes [18].

Despite the clear evidence that exercise training is a cornerstone in managing type 2 diabetes, individuals with the condition are among the least likely to engage in regular exercise. Among Canadian adults with type 2 diabetes, only 28% reported they were meeting public health and diabetes-specific exercise guidelines [19]. One of the most consistent predictors of exercise behaviour maintenance is self-efficacy, with confidence in the ability to exercise associated with greater adherence to exercise recommendations [20].

Blood glucose decreases during and after exercise for individuals with type 2 diabetes; however, this does not usually result in hypoglycaemia unless the individual is taking insulin or sulphonylureas. When hypoglycaemia occurs, the drug dose is usually in excess of the metabolic requirements and there are additional compounding problems (i.e. HAAF) [11]. Skeletal muscle insulin sensitivity is enhanced for up to 48 h following exercise increasing muscle glucose uptake and the risk of hypoglycaemia [21]. Increased insulin sensitivity may alter insulin and/or sulphonylurea requirements post-exercise for those with diabetes. Metformin use is postulated to increase the risk of lactic acidosis in those undertaking prolonged high-intensity exercise [22]. However, a recent review found this to be rare [23].

## Glucose Monitoring

It is recommended that all individuals with type 1 diabetes, and those with type 2 diabetes who are taking insulin and/or sulphonylureas, always check their glucose level two to three times prior to exercise to establish the direction of change in glucose. In addition, it is recommended that these individuals with type 1 diabetes also check their glucose level every 30 min during exercise and again after exercise. When initiating an exercise program or when implementing significant exercise program changes (e.g. increases in mode, intensity and duration), additional glucose testing is needed to understand the effects of the exercise on blood glucose and to avoid post-exercise hypoglycaemia. Establishing the glucose trend before, during and after exercise will educate and inform the exercise specialist regarding the effects of exercise on that individual. For people with type 2 diabetes managed with medications other than insulin or sulphonylureas (or with lifestyle alone), ongoing pre-exercise glucose testing is not generally necessary due to the low risk of hypoglycaemia.

Previous advice for monitoring glucose in the context of exercise has been provided in position/consensus statements targeting clinicians treating people with type 1 diabetes [24], type 2 diabetes [14, 25] and both [4] with these also communicated through other documents [26–28]. In response to the lack of bespoke resources for exercise specialists working with people with diabetes, an expert group was formed to develop the resources presented here. The group consisted of endocrinologists (PD, SM), accredited exercise physiologists (GT, SQ, JC) and a dietitian (RB). The group reviewed widespread sources including the position/consensus statements mentioned above and incorporated this information within the resources presented here. The aim of these resources is to provide general advice to exercise specialists regarding glucose management around the time of exercise undertaken by adults with diabetes. Furthermore, beyond these general guidelines, the level of fitness of the individual exercising and knowledge of their previous responses to exercise may enable further personalisation of the advice.

## General Recommendations

### Initial Review

All people with diabetes starting an exercise program should be reviewed to gather relevant information. This includes:

- Diabetes type;
- Medication regimen including any recent changes;
- Other relevant clinical data (e.g. fasting glucose level, blood pressure, heart rate, oxygen saturation);
- Co-morbidities; and.
- Factors that may specifically impact exercise program participation.

Healthcare professionals should consider the above information when considering an individual’s suitability to exercise. A screening tool may be useful to collect this information (e.g. Australian Pre-exercise Screening System; APSS [29], the Physical Activity Readiness Questionnaire for Everyone; PAR-Q+ [30] or the electronic Physical Activity Readiness Medical Examination; ePARmed-X+ [30]). Notably, algorithms associated with these tools are often overly conservative for people with diabetes resulting in excessive referrals for medical clearance [4]. The American Diabetes Association Position Statement states that pre-exercise medical clearance is not necessary for asymptomatic individuals receiving diabetes care consistent with guidelines if the intention is to exercise at a low- or moderate-intensity (e.g. not exceeding the demands of brisk walking or everyday living) [4]. A doctor should be consulted when co-morbidities, medications or the history of glucose control may complicate the introduction of an exercise program. In these cases, continued two-way communication between the doctor and exercise specialist working with the individual is essential. During the exercise program, the exercise specialist should regularly assess the individual’s current health status and identify any new symptoms or issues that arise.

Certain types of exercise are contraindicated when any of the following conditions are present: autonomic neuropathy, peripheral neuropathy, retinopathy, chest pain/discomfort, hypertension, nephropathy and hypoglycaemic unawareness. Identification of any of these during the screening process should prompt referral to a doctor for consideration of the advisable exercise types.

### Training of Exercise Specialists

People with diabetes may need to seek the assistance of an exercise specialist for guidance and/or supervised training. However, there are various levels of educational standards and qualifications in the exercise and fitness industries that can make it difficult to understand who is appropriately qualified to provide guidance. For example, in Australia, a person can attain a certificate in fitness (Certificate 3 in Fitness) in a short period of time and provide a personal training service. By comparison, in Australia, accredited exercise physiologists complete at least 4 years of university study with 500 h of exercise practicum. Professional standards of accredited exercise physiologists include detailed knowledge of physiology, pathophysiology and exercise training for diabetes. It is recommended that exercise prescription and delivery for people with diabetes be under the supervision of an accredited exercise physiologist or exercise specialist with similar knowledge and training (e.g. physiotherapist/physical therapist, clinical exercise physiologist in the USA, kinesiologist in Canada and biokinetisist in South Africa). Given the diversity of knowledge and experience in exercise specialists who may be training people with diabetes, it is essential that guidelines and resources such as those provided here are available to decrease the risk of an untoward event occurring during exercise training.

### Checklist Prior to Exercise Session

The following information should be obtained from the person prior to starting exercise:

- Timing, amount and type of previous food intake;
- Medications administered that would still be active (e.g. Lantus insulin administered the night prior would still be active the following day);
- Glucose level and trend prior to exercise (preference to use person’s own monitor to promote self-management). Two to three pre-exercise glucose measurements are recommended. Determine whether there has been severe hypoglycaemia within the past 24 h (i.e. hypoglycaemia that required assistance from another individual to treat). Note that for people with type 2 diabetes managed with medications other than insulin or sulphonylureas (or with lifestyle alone), ongoing pre-exercise glucose testing is not generally necessary due to the low risk of hypoglycaemia;
- Assessment of current health status and any new symptoms.

These details, together with the expected type, duration and intensity of the planned exercise session, will need to be known to appropriately monitor the person during the exercise session. Table 1 contains additional considerations and recommendations based on findings from the checklist.

**Table 1:** Additional considerations for people with diabetes exercising. A distinct PDF of this table can be viewed in Additional file 6

Every person with diabetes is different, tailor the exercise plan to meet individual needs.	
Assess the presence and severity of diabetes complications.	
If previous foot or nerve problems check feet for blisters and ulcers before and after exercise.	
Individuals with foot ulcers should avoid weight-bearing exercise that puts pressure on foot wounds.	
When beginning or modifying an exercise program, monitor glucose for several hours before and after exercise to observe the trend.	
Use of continuous glucose monitoring (via a transcutaneous sensor) provides greater detail regarding glucose changes, allowing finely-tuned medication adjustment.	
Hypoglycaemia is the main risk for people with diabetes that exercise. It may lead to a loss of consciousness and a diabetic coma, that is life threatening.	
For a person with type 1 diabetes about to exercise at a high intensity, a small correction insulin dose is recommended if glucose is > 6.9 mmol/L. This dose can be then taken away from next meal bolus.	
People with diabetes should consider exercising with a partner to assist in the detection of hypoglycaemia.	
Be aware of the timing of medication administration; in particular, be aware of insulin action profiles (e.g. for short/rapid-acting vs long-acting insulins).	
Consider effects of other medications: e.g. diuretics—fluid balance. e.g. beta-blockers—attenuate heart rate response to exercise; may mask hypoglycaemia symptoms of palpitations/racing heart. e.g. sodium-glucose co-transporter-2 (SGLT2) inhibitors—may cause severe acidosis with relatively normal glucose levels [31]. If feeling unwell after starting an SGLT2 inhibitor, postpone exercise and seek medical review.	
A person with type 1 diabetes taking an SGLT-inhibitor must be able to check ketones due to risk of ketosis including euglycaemic ketosis [31].	
Awareness of the 15 min delay between a blood glucose reading and a continuous glucose monitor’s (CGM’s) interstitial reading is important when planning exercise, especially when glucose level is low (e.g. a hypoglycaemic event has been treated and the blood glucose level is 5.5 mmol/L but the CGM may be measuring 4.5 mmol/L due to the delay).	
Diabetes may lead to cardiac autonomic dysfunction and a blunted heart rate and blood pressure response to exercise. Therefore, additional monitoring of blood pressure and the use of a rating of perceived exertion (RPE) to monitor exercise intensity may be needed.	
Insulin sensitivity varies diurnally, therefore different glucose responses may be observed with the same exercise undertaken at different times of the day.	
One of the safest times to exercise with the lowest variation in glucose response to exercise (i.e. easier to predict) is in the morning before breakfast (dependent on glucose level).	
A person with a glucose level frequently within the red area of the Action Plan should be reviewed by a Diabetes Healthcare Professional.	
Individuals with retinopathy should avoid higher intensity aerobic and resistance exercises (with large increases in systolic blood pressure), head-down activities, jumping or jarring activities. These all increase haemorrhage risk.	
Appropriate fluid intake is necessary to minimize dehydration and risk of heat stress. Increasing fluid intake is important when the glucose level is high.	

An important issue to consider is that trained individuals with diabetes have greater reductions in glucose during aerobic exercise compared with those who have reduced physical fitness [32]. Possible explanations for this include exercise training-induced effects on insulin sensitivity [33], glucose transporters [34], glucagon [35] or the use of glucagon-like peptide-1 [36] and/or dipeptidyl peptidase–4 [36, 37]. Another factor may be that with increased fitness people with diabetes are able to exercise at a greater workload, which may contribute to increased insulin sensitivity and greater glucose utilization [32].

## Guidelines for Starting or Continuing Exercise Based on Glucose Levels

**Fig. 1:**
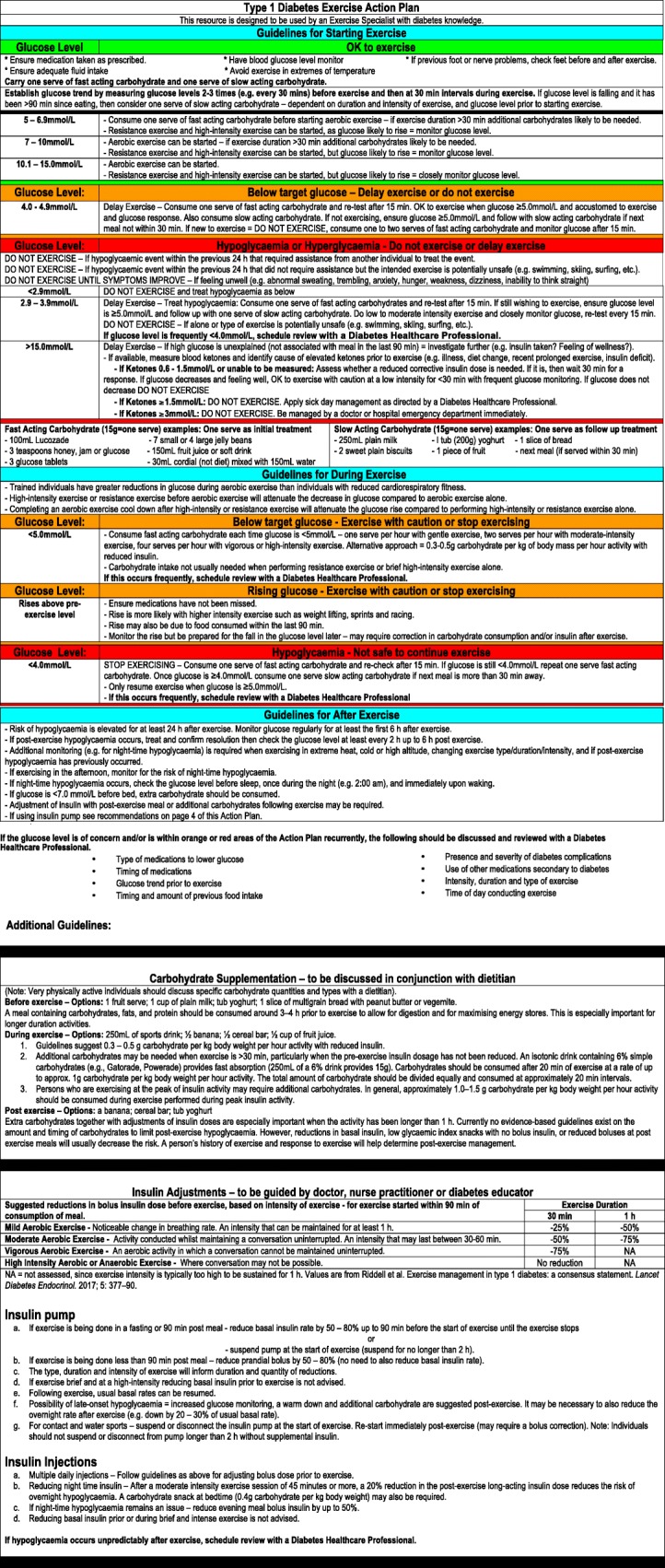
Type 1 Diabetes Exercise Action Plan. A distinct PDF of this figure can be viewed in Additional file 1

**Fig. 2:**
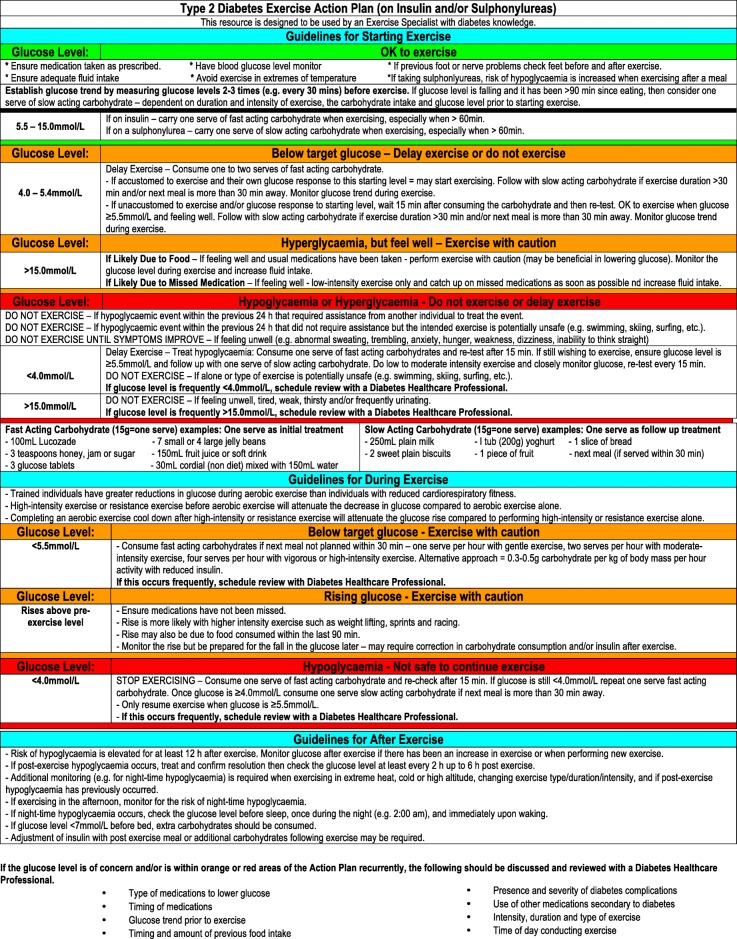
Type 2 Diabetes Exercise Action Plan (on insulin and/or sulphonylureas). A distinct PDF of this figure can be viewed in Additional file 2

**Fig. 3:**
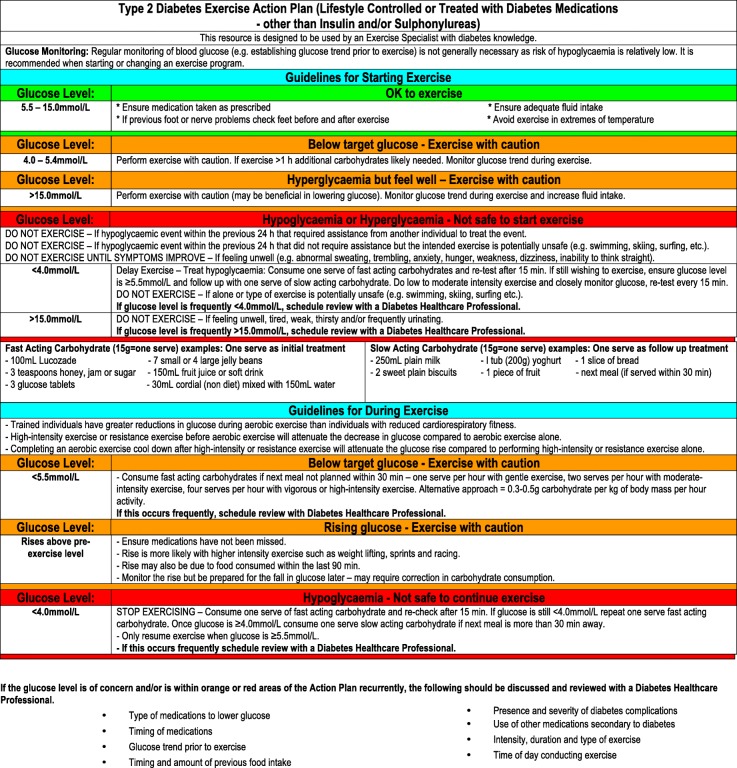
Type 2 Diabetes Exercise Action Plan (lifestyle controlled or treated with diabetes medications—other than insulin and/or sulphonylureas). A distinct PDF of this figure can be viewed in Additional file 3

**Fig. 4:**
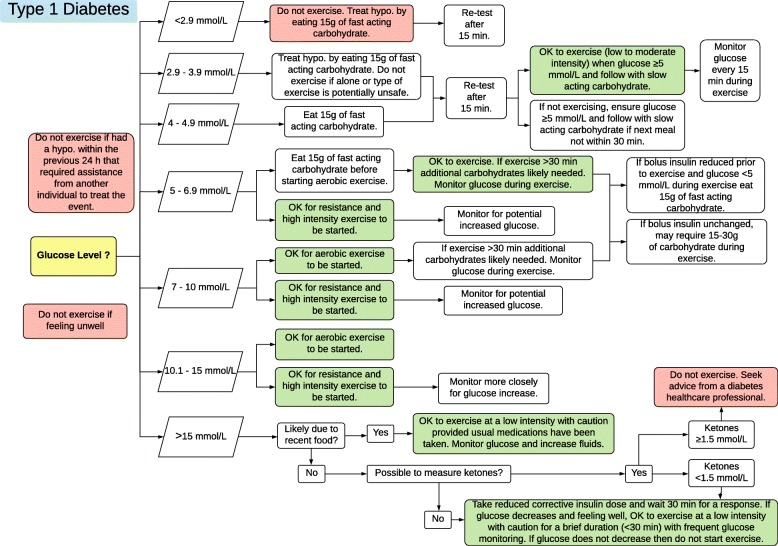
Type 1 Diabetes Decision Tree: for use by an exercise specialist with diabetes knowledge. A distinct PDF of this figure can be viewed in Additional file 4

**Fig. 5:**
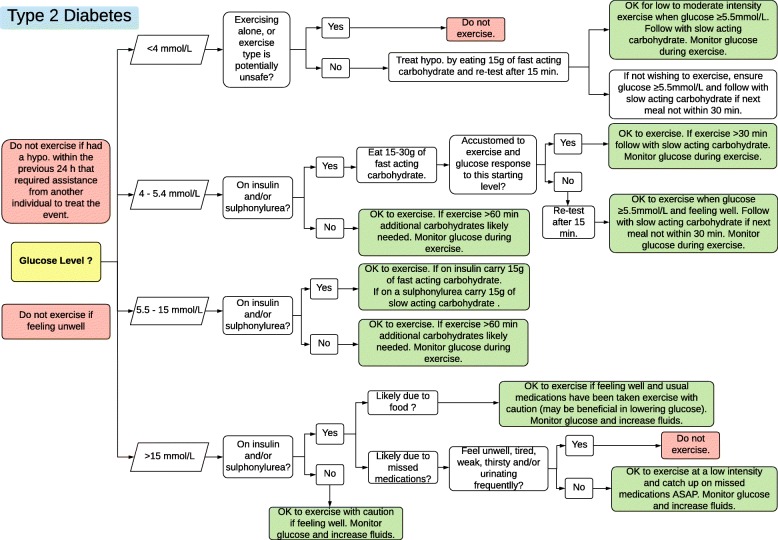
Type 2 Diabetes Decision Tree: for use by an exercise specialist with diabetes knowledge. A distinct PDF of this figure can be viewed in Additional file 5

Figures 1, 2 and 3 contain guidelines using a traffic light approach to assist exercise specialists with clinical decision-making regarding people with diabetes starting or continuing exercise based on glucose levels and other factors. These Action Plans will be individually guided by the exercise specialist, in consultation with the individual. Figures 4 and 5 contain simplified flow charts summarizing the guidelines. These resources refer to a Diabetes Healthcare Professional who is a clinician with appropriate qualifications to understand the interactions between medications, glucose levels, carbohydrate intake and co-morbidities. For example, in Australia, this includes doctors, nurse practitioners, credentialed diabetes educators, accredited exercise physiologists, physiotherapists and accredited practicing dietitians.

### Type 1 Diabetes

For people with type 1 diabetes, hypoglycaemia during and for up to 24 h following exercise are usually the main risks. The following recommendations are from a recent international consensus statement on type 1 diabetes and exercise [24].

Exercise is contraindicated if glucose has been < 2.9 mmol/L or if a hypoglycaemic event that required assistance from another person to treat the event within the previous 24 h. These situations significantly increase the risk of a more serious hypoglycaemic episode occurring during exercise. If glucose is between 2.9 and 3.9 mmol/L, exercise should not commence until the hypoglycaemia is treated. However, even after treatment, if starting glucose was 2.9–3.9 mmol/L, exercise should be avoided if alone, or the type of exercise is potentially unsafe (e.g. swimming, skiing, surfing, rock climbing etc.). The Action Plan (Fig. 1) and flow chart (Fig. 4) provide more guidance for various scenarios.

Initial hypoglycaemia treatment involves consuming one serve (15 g) of fast-acting carbohydrate and re-checking glucose after 15 min. Another serve of fast-acting carbohydrate should be administered each 15 min if glucose remains < 4.0 mmol/L. After initial treatment, monitoring is advised for clinical features of hypoglycaemia such as abnormal sweating, trembling, anxiety, hunger, weakness, dizziness, inability to think straight and tingling sensations in the mouth and/or fingers. If still wishing to exercise, ensure glucose is ≥ 5.0 mmol/L before beginning the exercise and follow up with one serve of slow-acting carbohydrate. Closely monitor glucose levels by re-testing every 15 min. If not wishing to exercise, ensure glucose is ≥ 5.0 mmol/L and follow up with slow-acting carbohydrate if the next meal is not within 30 min. Figure 1 provides examples of foods that are classified as fast- and slow-acting carbohydrate.

A number of additional factors can increase the risk of hypoglycaemia during or after exercise including increased circulating insulin from the release of residual injected insulin, inadequate glucose production from the liver, individual fitness level, glycogen recovery, the mode, duration and intensity of exercise, the environment and the person’s hydration status [33, 38]. Figure 1 provides specific guidelines for monitoring glucose after exercise. An increased risk of night-time hypoglycaemia due to afternoon/evening exercise or changes in exercise (e.g. increased intensity/duration) should lead to more glucose surveillance. Measuring glucose before bed and setting an alarm to wake up and check blood glucose (e.g. 2.00 am) or using a continual glucose monitor with an alarm is recommended. If the glucose level is < 7 mmol/L before bed, additional carbohydrates should be consumed.

As mentioned previously, it is recommended that the glucose trend be established prior to exercising with two to three glucose measures. The glucose target for the start of exercise for a person with type 1 diabetes should be individualized based on the intended type, duration and intensity of exercise, when medications were used and food consumed, the trend in glucose and exercise experience. As a general guideline for most people intending to complete any type of exercise for around 1 h, a starting glucose between 7.0 and 10.0 mmol/L is recommended. Figures 1 and 4 provide advice for when the starting glucose is outside this range and for carbohydrate consumption during exercise.

Hyperglycaemia during or following exercise may be associated with ketosis (due to absolute or relative insulin insufficiency). A glucose level > 15.0 mmol/L is used as a threshold to investigate further. This includes assessing whether food has been consumed in the previous 90 min, if the person has had their usual insulin dose, whether they are feeling well and for the presence of ketones. If small to moderate levels of blood ketones are present (0.6–1.5 mmol/L) or if ketones can not be measured then the need for a reduced corrective insulin dose should be assessed. If this is needed, then glucose should be checked after 30 min and if it is decreasing and the person is feeling well then low-intensity, short duration (< 30 min) exercise can be started with caution. More substantial ketosis is an absolute contraindication and may require medical attention. The Action Plan provides specific recommendations for the possible scenarios based on these measures. A glucose level > 15.0 mmol/L should trigger extra surveillance of the person’s general feeling of wellness. Dehydration can result from frequent urination due to hyperglycaemia and may lead to symptoms of heat illness, especially when exercising. Remaining hydrated is especially important when the glucose level is high.

Elevations in blood glucose are more likely following high-intensity or resistance exercise [39, 40]. This is likely due a number of mechanisms including an increased stress response leading to hormones such as catecholamines inducing gluconeogenesis and glycogenolysis [41]. A prolonged aerobic cool down has been recommended to minimize glycaemic excursions [24].

### Type 2 Diabetes

The following recommendations for people with type 2 diabetes are consistent with a recent position statement from the American Diabetes Association. [4]

#### Type 2 Diabetes Treated with Insulin and/or Sulphonylureas

Hypoglycaemia during exercise, or for up to 12 h after exercise, is the main risk for individuals with type 2 diabetes taking insulin and/or sulfonylurea medication. Exercise is contraindicated if a person has had a hypoglycaemic event that required assistance from another person to treat the event within the previous 24 h, if feeling unwell or glucose is < 4.0 mmol/L and the intended exercise is being done alone or is potentially unsafe. The Action Plan (Fig. 2) and flow chart (Fig. 5) provide more guidance for various scenarios.

Glucose between 4.0 and 5.4 mmol/L may herald impending hypoglycaemia during exercise and warrants one to two serves of fast-acting carbohydrate. For individuals aware of their own response to exercise with this starting glucose level, this may be sufficient. For those new to exercise, with glucose between 4.0 and 5.4 mmol/L, exercise should not start and glucose monitoring should occur 15 min later. Once glucose is ≥ 5.5 mmol/L and the individual has no symptoms of feeling unwell, then exercise can start. Sulphonylureas are insulin secretagogues that increase the risk of hypoglycaemia during moderate to high-intensity exercise. Being aware of each person’s insulin/sulphonylurea action profile is important. People taking short/rapid/intermediate-acting insulin should avoid exercising when blood insulin is peaking.

As mentioned previously, it is recommended that the glucose trend be established prior to exercising with two to three glucose measures. If the glucose level is falling and it has been greater than 90 min since eating, then one serve of a slow-acting carbohydrate should be considered. This will be dependent on the duration and intensity of exercise, the carbohydrate intake and the glucose level prior to the start of exercise.

To prevent hypoglycaemia, the timing of exercise and/or medication administration and/or dose should be considered. If night-time hypoglycaemia is likely, check the glucose level before sleep, once during the night (e.g. 2:00 am) and immediately upon waking. If the glucose level is < 7 mmol/L before bed, additional carbohydrates should be consumed. If the glucose level is frequently within the red area of the Action Plan, a Diabetes Healthcare Professional should be consulted to review the factors that may be causing the sub-optimal glucose control.

The glucose target for the start of exercise for a person with type 2 diabetes treated with Insulin and/or Sulphonylureas is between 5.5 and 15.0 mmol/L. If exercise is longer than 1 h, additional carbohydrates are likely to be needed. If an individual has a glucose level > 15.0 mmol/L, assess whether it is due to inadequate insulin treatment, acute illness or infection or food intake. If the glucose level is measured within 2 h of eating or the previous food had a high glycaemic index, exercise may be beneficial in lowering the glucose. Extra fluid intake is advised if exercising with high glucose. If the high glucose is due to missed medications, exercise at a low intensity and ensure that the person catches up on the missed dose as soon as possible. If the high glucose level is due to acute illness or infection, postpone exercise.

#### Type 2 Diabetes (Lifestyle Controlled or Treated with Diabetes Medications Other than Insulin or Sulphonylureas)

The interaction of exercise with diabetes medications other than insulin and sulphonylureas has not been well studied [14, 25]. Drugs such as biguanides (e.g. metformin), thiazolidinediones (e.g. rosiglitazone), alpha-glucosidase inhibitors (e.g. acarbose), sodium-glucose transporter-2 (SGLT) inhibitors (e.g. dapagliflozin, empagliflozin) and glucagon-like peptide 1 (GLP-1) agonists (e.g. exenatide) are thought to have a minimal effect on increasing the risk of exercise-induced hypoglycaemia when used alone. However, these drugs can potentiate the hypoglycaemia effects of insulin and sulphonylureas. It is recommended that regular glucose monitoring is only necessary in individuals taking any of these medications when starting or changing an exercise program. When these medications are combined with a sulphonylurea and/or insulin, additional monitoring as per the insulin/sulphonylurea guidelines should be conducted.

To prevent hypoglycaemia, the timing of exercise and/or medication administration and/or dose may need to be considered. A doctor, nurse practitioner or diabetes educator should be consulted prior to changing medication dose. If the glucose level is frequently within the red area of the Action Plan, a Diabetes Healthcare Professional should be consulted to review the factors that may be causing the sub-optimal glucose control. The Action Plan (Fig. 3) and flow chart (Fig. 5) provide more guidance for various scenarios.

## Additional Considerations for People with Diabetes

The effect of diabetes on the response to exercise is dependent on many variables. Table 1 provides a number of considerations that will improve the safety of exercise for an individual with diabetes.

## Conclusion

In summary, exercise has major and widespread benefits for people with diabetes. For most people with diabetes, exercise is safe and beneficial. Avoiding hypoglycaemia and circumstances which may promote ketosis is important. Knowledge of an individual’s previous response to exercise will assist in implementing these guidelines. Glucose monitoring before, during and after exercise may be needed to inform strategies and maintain stable and safe levels. Providing exercise guidance and/or training an individual with diabetes can be challenging and requires advanced knowledge and experience. The resources presented here are provided to maximize the safety of exercise training for individuals with diabetes and to realize the potential health benefits of exercise.

## Additional files

Additional file 1: Type 1 Diabetes Exercise Action Plan. (PDF 162 kb)

Additional file 2: Type 2 Diabetes Exercise Action Plan (on Insulin and/or Sulphonylureas). (PDF 114 kb)

Additional file 3: Type 2 Diabetes Exercise Action Plan (Lifestyle Controlled or Treated with Diabetes Medications - other than Insulin and/or Sulphonylureas). (PDF 98 kb)

Additional file 4: Type 1 Diabetes. (PDF 49 kb)

Additional file 5: Type 2 Diabetes. (PDF 48 kb)

Additional file 6: Additional Considerations for People with Diabetes Exercising. (PDF 78 kb)

## Abbreviations

APSS: Australian Pre-exercise Screening System
ePARmed-X+: Electronic Physical Activity Readiness Medical Examination
GLP-1: Glucagon-like peptide 1
hypo: Hypoglycaemic event
MDI: Multiple daily injections
PAR-Q+: Physical Activity Readiness Questionnaire for Everyone
RPE: Rating of perceived exertion
SGLT2: Sodium-glucose co-transporter-2
