# Supplementary material for: Resources to Guide Exercise Specialists Managing Adults with Diabetes
Source: Sports Med Open. 2019 Jun 3;5:20. doi: 10.1186/s40798-019-0192-1 (PMC6546780; doi:10.1186/s40798-019-0192-1)
Supplement: Supplementary file 1 — Type 1 Diabetes Exercise Action Plan. (PDF 162 kb) [file 40798_2019_192_MOESM1_ESM.pdf]

Type 1 Diabetes Exercise Action Plan

This resource is designed to be used by an Exercise Specialist with diabetes knowledge.

Guidelines for Starting Exercise

Glucose Level	OK to exercise
* Ensure medication taken as prescribed. * Have blood glucose level monitor * If previous foot or nerve problems, check feet before and after exercise.<br>* Ensure adequate fluid intake * Avoid exercise in extremes of temperature<br>**Carry one serve of fast acting carbohydrate and one serve of slow acting carbohydrate.**	
**Establish glucose trend by measuring glucose levels 2-3 times (e.g. every 30 mins) before exercise and then at 30 min intervals during exercise.** If glucose level is falling and it has been >90 min since eating, then consider one serve of slow acting carbohydrate – dependent on duration and intensity of exercise, and glucose level prior to starting exercise.	
**5 – 6.9mmol/L**	- Consume one serve of fast acting carbohydrate before starting aerobic exercise – if exercise duration >30 min additional carbohydrates likely to be needed.<br>- Resistance exercise and high-intensity exercise can be started, as glucose likely to rise = monitor glucose level.
**7 – 10mmol/L**	- Aerobic exercise can be started – if exercise duration >30 min additional carbohydrates likely to be needed.<br>- Resistance exercise and high-intensity exercise can be started, but glucose likely to rise = monitor glucose level.
**10.1 – 15.0mmol/L**	- Aerobic exercise can be started.<br>- Resistance exercise and high-intensity exercise can be started, but glucose likely to rise = closely monitor glucose level.

Glucose Level:	Below target glucose – Delay exercise or do not exercise
**4.0 - 4.9mmol/L**	Delay Exercise – Consume one serve of fast acting carbohydrate and re-test after 15 min. OK to exercise when glucose ≥5.0mmol/L and accustomed to exercise and glucose response. Also consume slow acting carbohydrate. If not exercising, ensure glucose ≥5.0mmol/L and follow with slow acting carbohydrate if next meal not within 30 min. If new to exercise = DO NOT EXERCISE, consume one to two serves of fast acting carbohydrate and monitor glucose after 15 min.

Glucose Level:	Hypoglycaemia or Hyperglycaemia - Do not exercise or delay exercise
DO NOT EXERCISE – If hypoglycaemic event within the previous 24 h that required assistance from another individual to treat the event.<br>DO NOT EXERCISE – If hypoglycaemic event within the previous 24 h that did not require assistance but the intended exercise is potentially unsafe (e.g. swimming, skiing, surfing, etc.).<br>DO NOT EXERCISE UNTIL SYMPTOMS IMPROVE – If feeling unwell (e.g. abnormal sweating, trembling, anxiety, hunger, weakness, dizziness, inability to think straight)	
**<2.9mmol/L**	DO NOT EXERCISE and treat hypoglycaemia as below
**2.9 – 3.9mmol/L**	Delay Exercise – Treat hypoglycaemia: Consume one serve of fast acting carbohydrates and re-test after 15 min. If still wishing to exercise, ensure glucose level is ≥5.0mmol/L and follow up with one serve of slow acting carbohydrate. Do low to moderate intensity exercise and closely monitor glucose, re-test every 15 min.<br>DO NOT EXERCISE – If alone or type of exercise is potentially unsafe (e.g. swimming, skiing, surfing, etc.).<br>**If glucose level is frequently <4.0mmol/L, schedule review with a Diabetes Healthcare Professional.**
**>15.0mmol/L**	Delay Exercise – If high glucose is unexplained (not associated with meal in the last 90 min) = investigate further (e.g. insulin taken? Feeling of wellness?).<br>- If available, measure blood ketones and identify cause of elevated ketones prior to exercise (e.g. illness, diet change, recent prolonged exercise, insulin deficit).<br>**- If Ketones 0.6 - 1.5mmol/L or unable to be measured:** Assess whether a reduced corrective insulin dose is needed. If it is, then wait 30 min for a response. If glucose decreases and feeling well, OK to exercise with caution at a low intensity for <30 min with frequent glucose monitoring. If glucose does not decrease DO NOT EXERCISE<br>**- If Ketones ≥1.5mmol/L:** DO NOT EXERCISE. Apply sick day management as directed by a Diabetes Healthcare Professional.<br>**- If Ketones ≥3mmol/L:** DO NOT EXERCISE. Be managed by a doctor or hospital emergency department immediately.

**Fast Acting Carbohydrate (15g=one serve) examples: One serve as initial treatment**	**Slow Acting Carbohydrate (15g=one serve) examples: One serve as follow up treatment**
- 100mL Lucozade<br>- 7 small or 4 large jelly beans<br>- 3 teaspoons honey, jam or glucose<br>- 150mL fruit juice or soft drink<br>- 3 glucose tablets<br>- 30mL cordial (not diet) mixed with 150mL water	- 250mL plain milk<br>- I tub (200g) yoghurt<br>- 1 slice of bread<br>- 2 sweet plain biscuits<br>- 1 piece of fruit<br>- next meal (if served within 30 min)

Guidelines for During Exercise	
- Trained individuals have greater reductions in glucose during aerobic exercise than individuals with reduced cardiorespiratory fitness.<br>- High-intensity exercise or resistance exercise before aerobic exercise will attenuate the decrease in glucose compared to aerobic exercise alone.<br>- Completing an aerobic exercise cool down after high-intensity or resistance exercise will attenuate the glucose rise compared to performing high-intensity or resistance exercise alone.	
**Glucose Level:**	**Below target glucose - Exercise with caution or stop exercising**
**<5.0mmol/L**	- Consume fast acting carbohydrate each time glucose is <5mmol/L – one serve per hour with gentle exercise, two serves per hour with moderate-intensity exercise, four serves per hour with vigorous or high-intensity exercise. Alternative approach = 0.3-0.5g carbohydrate per kg of body mass per hour activity with reduced insulin.<br>- Carbohydrate intake not usually needed when performing resistance exercise or brief high-intensity exercise alone.<br>**If this occurs frequently, schedule review with a Diabetes Healthcare Professional.**
**Glucose Level:**	**Rising glucose - Exercise with caution or stop exercising**
**Rises above pre-exercise level**	- Ensure medications have not been missed.<br>- Rise is more likely with higher intensity exercise such as weight lifting, sprints and racing.<br>- Rise may also be due to food consumed within the last 90 min.<br>- Monitor the rise but be prepared for the fall in the glucose level later – may require correction in carbohydrate consumption and/or insulin after exercise.
**Glucose Level:**	**Hypoglycaemia - Not safe to continue exercise**
**<4.0mmol/L**	STOP EXERCISING – Consume one serve of fast acting carbohydrate and re-check after 15 min. If glucose is still <4.0mmol/L repeat one serve fast acting carbohydrate. Once glucose is ≥4.0mmol/L consume one serve slow acting carbohydrate if next meal is more than 30 min away.<br>- Only resume exercise when glucose is ≥5.0mmol/L.<br>**- If this occurs frequently, schedule review with a Diabetes Healthcare Professional**

Guidelines for After Exercise

- Risk of hypoglycaemia is elevated for at least 24 h after exercise. Monitor glucose regularly for at least the first 6 h after exercise.
- If post-exercise hypoglycaemia occurs, treat and confirm resolution then check the glucose level at least every 2 h up to 6 h post exercise.
- Additional monitoring (e.g. for night-time hypoglycaemia) is required when exercising in extreme heat, cold or high altitude, changing exercise type/duration/intensity, and if post-exercise hypoglycaemia has previously occurred.
- If exercising in the afternoon, monitor for the risk of night-time hypoglycaemia.
- If night-time hypoglycaemia occurs, check the glucose level before sleep, once during the night (e.g. 2:00 am), and immediately upon waking.
- If glucose is <7.0 mmol/L before bed, extra carbohydrate should be consumed.
- Adjustment of insulin with post-exercise meal or additional carbohydrates following exercise may be required.
- If using insulin pump see recommendations on page 4 of this Action Plan.

If the glucose level is of concern and/or is within orange or red areas of the Action Plan recurrently, the following should be discussed and reviewed with a Diabetes Healthcare Professional.

- Type of medications to lower glucose
- Timing of medications
- Glucose trend prior to exercise
- Timing and amount of previous food intake
- Presence and severity of diabetes complications
- Use of other medications secondary to diabetes
- Intensity, duration and type of exercise
- Time of day conducting exercise

Additional Guidelines:

Carbohydrate Supplementation – to be discussed in conjunction with dietitian

(Note: Very physically active individuals should discuss specific carbohydrate quantities and types with a dietitian).

Before exercise – Options: 1 fruit serve; 1 cup of plain milk; tub yoghurt; 1 slice of multigrain bread with peanut butter or vegemite.

A meal containing carbohydrates, fats, and protein should be consumed around 3–4 h prior to exercise to allow for digestion and for maximising energy stores. This is especially important for longer duration activities.

During exercise – Options: 250mL of sports drink; ½ banana; ½ cereal bar; ½ cup of fruit juice.

1. Guidelines suggest 0.3 – 0.5 g carbohydrate per kg body weight per hour activity with reduced insulin.
2. Additional carbohydrates may be needed when exercise is >30 min, particularly when the pre-exercise insulin dosage has not been reduced. An isotonic drink containing 6% simple carbohydrates (e.g., Gatorade, Powerade) provides fast absorption (250mL of a 6% drink provides 15g). Carbohydrates should be consumed after 20 min of exercise at a rate of up to approx. 1g carbohydrate per kg body weight per hour activity. The total amount of carbohydrate should be divided equally and consumed at approximately 20 min intervals.
3. Persons who are exercising at the peak of insulin activity may require additional carbohydrates. In general, approximately 1.0–1.5 g carbohydrate per kg body weight per hour activity should be consumed during exercise performed during peak insulin activity.

Post exercise – Options: a banana; cereal bar; tub yoghurt

Extra carbohydrates together with adjustments of insulin doses are especially important when the activity has been longer than 1 h. Currently no evidence-based guidelines exist on the amount and timing of carbohydrates to limit post-exercise hypoglycaemia. However, reductions in basal insulin, low glycaemic index snacks with no bolus insulin, or reduced boluses at post exercise meals will usually decrease the risk. A person’s history of exercise and response to exercise will help determine post-exercise management.

Additional Guidelines:

Insulin Adjustments – to be guided by doctor, nurse practitioner or diabetes educator

Suggested reductions in bolus insulin dose before exercise, based on intensity of exercise - for exercise started within 90 min of consumption of meal.	Exercise Duration	
	**30 min**	**1 h**
**Mild Aerobic Exercise -** Noticeable change in breathing rate. An intensity that can be maintained for at least 1 h.	-25%	-50%
**Moderate Aerobic Exercise -** Activity conducted whilst maintaining a conversation uninterrupted. An intensity that may last between 30-60 min.	-50%	-75%
**Vigorous Aerobic Exercise -** An aerobic activity in which a conversation cannot be maintained uninterrupted.	-75%	NA
**High Intensity Aerobic or Anaerobic Exercise -** Where conversation may not be possible.	No reduction	NA

NA = not assessed, since exercise intensity is typically too high to be sustained for 1 h. Values are from Riddell et al. Exercise management in type 1 diabetes: a consensus statement. *Lancet Diabetes Endocrinol.* 2017; 5: 377–90.

Insulin pump

a. If exercise is being done in a fasting or 90 min post meal - reduce basal insulin rate by 50 – 80% up to 90 min before the start of exercise until the exercise stops
or
- suspend pump at the start of exercise (suspend for no longer than 2 h).
b. If exercise is being done less than 90 min post meal – reduce prandial bolus by 50 – 80% (no need to also reduce basal insulin rate).
c. The type, duration and intensity of exercise will inform duration and quantity of reductions.
d. If exercise brief and at a high-intensity reducing basal insulin prior to exercise is not advised.
e. Following exercise, usual basal rates can be resumed.
f. Possibility of late-onset hypoglycaemia = increased glucose monitoring, a warm down and additional carbohydrate are suggested post-exercise. It may be necessary to also reduce the overnight rate after exercise (e.g. down by 20 – 30% of usual basal rate).
g. For contact and water sports – suspend or disconnect the insulin pump at the start of exercise. Re-start immediately post-exercise (may require a bolus correction). Note: Individuals should not suspend or disconnect from pump longer than 2 h without supplemental insulin.

Insulin Injections

a. Multiple daily injections – Follow guidelines as above for adjusting bolus dose prior to exercise.
b. Reducing night time insulin – After a moderate intensity exercise session of 45 minutes or more, a 20% reduction in the post-exercise long-acting insulin dose reduces the risk of overnight hypoglycaemia. A carbohydrate snack at bedtime (0.4g carbohydrate per kg body weight) may also be required.
c. If night-time hypoglycaemia remains an issue – reduce evening meal bolus insulin by up to 50%.
d. Reducing basal insulin prior or during brief and intense exercise is not advised.

If hypoglycaemia occurs unpredictably after exercise, schedule review with a Diabetes Healthcare Professional.
