# Supplementary material for: Resources to Guide Exercise Specialists Managing Adults with Diabetes
Source: Sports Med Open. 2019 Jun 3;5:20. doi: 10.1186/s40798-019-0192-1 (PMC6546780; doi:10.1186/s40798-019-0192-1)
Supplement: Supplementary file 2 — Type 2 Diabetes Exercise Action Plan (on Insulin and/or Sulphonylureas). (PDF 114 kb) [file 40798_2019_192_MOESM2_ESM.pdf]

Type 2 Diabetes Exercise Action Plan (on Insulin and/or Sulphonylureas)

This resource is designed to be used by an Exercise Specialist with diabetes knowledge.

Guidelines for Starting Exercise

Glucose Level:	OK to exercise

* Ensure medication taken as prescribed. * Have blood glucose level monitor * If previous foot or nerve problems check feet before and after exercise.
* Ensure adequate fluid intake * Avoid exercise in extremes of temperature *If taking sulphonlyureas, risk of hypoglycaemia is increased when exercising after a meal

Establish glucose trend by measuring glucose levels 2-3 times (e.g. every 30 mins) before exercise. If glucose level is falling and it has been >90 min since eating, then consider one serve of slow acting carbohydrate – dependent on duration and intensity of exercise, the carbohydrate intake and glucose level prior to starting exercise.

**5.5 – 15.0mmol/L**	If on insulin – carry one serve of fast acting carbohydrate when exercising, especially when > 60min.<br>If on a sulphonylurea – carry one serve of slow acting carbohydrate when exercising, especially when > 60min.

Glucose Level:	Below target glucose – Delay exercise or do not exercise
**4.0 – 5.4mmol/L**	Delay Exercise – Consume one to two serves of fast acting carbohydrate.<br>- If accustomed to exercise and their own glucose response to this starting level = may start exercising. Follow with slow acting carbohydrate if exercise duration >30 min and/or next meal is more than 30 min away. Monitor glucose trend during exercise.<br>- If unaccustomed to exercise and/or glucose response to starting level, wait 15 min after consuming the carbohydrate and then re-test. OK to exercise when glucose ≥5.5mmol/L and feeling well. Follow with slow acting carbohydrate if exercise duration >30 min and/or next meal is more than 30 min away. Monitor glucose trend during exercise.

Glucose Level:	Hyperglycaemia, but feel well – Exercise with caution
**>15.0mmol/L**	**If Likely Due to Food** – If feeling well and usual medications have been taken - perform exercise with caution (may be beneficial in lowering glucose). Monitor the glucose level during exercise and increase fluid intake.<br>**If Likely Due to Missed Medication** – If feeling well - low-intensity exercise only and catch up on missed medications as soon as possible nd increase fluid intake.

Glucose Level:	Hypoglycaemia or Hyperglycaemia - Do not exercise or delay exercise

DO NOT EXERCISE – If hypoglycaemic event within the previous 24 h that required assistance from another individual to treat the event.
DO NOT EXERCISE – If hypoglycaemic event within the previous 24 h that did not require assistance but the intended exercise is potentially unsafe (e.g. swimming, skiing, surfing, etc.).
DO NOT EXERCISE UNTIL SYMPTOMS IMPROVE – If feeling unwell (e.g. abnormal sweating, trembling, anxiety, hunger, weakness, dizziness, inability to think straight)

**<4.0mmol/L**	Delay Exercise – Treat hypoglycaemia: Consume one serve of fast acting carbohydrates and re-test after 15 min. If still wishing to exercise, ensure glucose level is ≥5.5mmol/L and follow up with one serve of slow acting carbohydrate. Do low to moderate intensity exercise and closely monitor glucose, re-test every 15 min.<br>DO NOT EXERCISE – If alone or type of exercise is potentially unsafe (e.g. swimming, skiing, surfing, etc.).<br>**If glucose level is frequently <4.0mmol/L, schedule review with a Diabetes Healthcare Professional.**
**>15.0mmol/L**	DO NOT EXERCISE – If feeling unwell, tired, weak, thirsty and/or frequently urinating.<br>**If glucose level is frequently >15.0mmol/L, schedule review with a Diabetes Healthcare Professional.**

Fast Acting Carbohydrate (15g=one serve) examples: One serve as initial treatment
- 100mL Lucozade
- 7 small or 4 large jelly beans
- 3 teaspoons honey, jam or sugar
- 150mL fruit juice or soft drink
- 3 glucose tablets
- 30mL cordial (non diet) mixed with 150mL water

Slow Acting Carbohydrate (15g=one serve) examples: One serve as follow up treatment
- 250mL plain milk
- I tub (200g) yoghurt
- 1 slice of bread
- 2 sweet plain biscuits
- 1 piece of fruit
- next meal (if served within 30 min)

Guidelines for During Exercise

- Trained individuals have greater reductions in glucose during aerobic exercise than individuals with reduced cardiorespiratory fitness.
- High-intensity exercise or resistance exercise before aerobic exercise will attenuate the decrease in glucose compared to aerobic exercise alone.
- Completing an aerobic exercise cool down after high-intensity or resistance exercise will attenuate the glucose rise compared to performing high-intensity or resistance exercise alone.

Glucose Level:	Below target glucose - Exercise with caution
**<5.5mmol/L**	- Consume fast acting carbohydrates if next meal not planned within 30 min – one serve per hour with gentle exercise, two serves per hour with moderate-intensity exercise, four serves per hour with vigorous or high-intensity exercise. Alternative approach = 0.3-0.5g carbohydrate per kg of body mass per hour activity with reduced insulin.<br>**If this occurs frequently, schedule review with Diabetes Healthcare Professional.**
**Glucose Level:**	**Rising glucose - Exercise with caution**
**Rises above pre-exercise level**	- Ensure medications have not been missed.<br>- Rise is more likely with higher intensity exercise such as weight lifting, sprints and racing.<br>- Rise may also be due to food consumed within the last 90 min.<br>- Monitor the rise but be prepared for the fall in the glucose later – may require correction in carbohydrate consumption and/or insulin after exercise.

Glucose Level:	Hypoglycaemia - Not safe to continue exercise
**<4.0mmol/L**	STOP EXERCISING – Consume one serve of fast acting carbohydrate and re-check after 15 min. If glucose is still <4.0mmol/L repeat one serve fast acting carbohydrate. Once glucose is ≥4.0mmol/L consume one serve slow acting carbohydrate if next meal is more than 30 min away.<br>- Only resume exercise when glucose is ≥5.5mmol/L.<br>**- If this occurs frequently, schedule review with a Diabetes Healthcare Professional.**

Guidelines for After Exercise

- Risk of hypoglycaemia is elevated for at least 12 h after exercise. Monitor glucose after exercise if there has been an increase in exercise or when performing new exercise.
- If post-exercise hypoglycaemia occurs, treat and confirm resolution then check the glucose level at least every 2 h up to 6 h post exercise.
- Additional monitoring (e.g. for night-time hypoglycaemia) is required when exercising in extreme heat, cold or high altitude, changing exercise type/duration/intensity, and if post-exercise hypoglycaemia has previously occurred.
- If exercising in the afternoon, monitor for the risk of night-time hypoglycaemia.
- If night-time hypoglycaemia occurs, check the glucose level before sleep, once during the night (e.g. 2:00 am), and immediately upon waking.
- If glucose level <7mmol/L before bed, extra carbohydrates should be consumed.
- Adjustment of insulin with post exercise meal or additional carbohydrates following exercise may be required.

If the glucose level is of concern and/or is within orange or red areas of the Action Plan recurrently, the following should be discussed and reviewed with a Diabetes Healthcare Professional.

- Type of medications to lower glucose
- Timing of medications
- Glucose trend prior to exercise
- Timing and amount of previous food intake
- Presence and severity of diabetes complications
- Use of other medications secondary to diabetes
- Intensity, duration and type of exercise
- Time of day conducting exercise
