# Supplementary material for: Resources to Guide Exercise Specialists Managing Adults with Diabetes
Source: Sports Med Open. 2019 Jun 3;5:20. doi: 10.1186/s40798-019-0192-1 (PMC6546780; doi:10.1186/s40798-019-0192-1)
Supplement: Supplementary file 3 — Type 2 Diabetes Exercise Action Plan (Lifestyle Controlled or Treated with Diabetes Medications - other than Insulin and/or Sulphonylureas). (PDF 98 kb) [file 40798_2019_192_MOESM3_ESM.pdf]

Type 2 Diabetes Exercise Action Plan (Lifestyle Controlled or Treated with Diabetes Medications - other than Insulin and/or Sulphonylureas)

This resource is designed to be used by an Exercise Specialist with diabetes knowledge.

Glucose Monitoring: Regular monitoring of blood glucose (e.g. establishing glucose trend prior to exercise) is not generally necessary as risk of hypoglycaemia is relatively low. It is recommended when starting or changing an exercise program.

Guidelines for Starting Exercise

Glucose Level:	OK to exercise
**5.5 – 15.0mmol/L**	* Ensure medication taken as prescribed<br>* If previous foot or nerve problems check feet before and after exercise<br>* Ensure adequate fluid intake<br>* Avoid exercise in extremes of temperature

Glucose Level:	Below target glucose - Exercise with caution
**4.0 – 5.4mmol/L**	Perform exercise with caution. If exercise >1 h additional carbohydrates likely needed. Monitor glucose trend during exercise.
**Glucose Level:**	**Hyperglycaemia but feel well – Exercise with caution**
**>15.0mmol/L**	Perform exercise with caution (may be beneficial in lowering glucose). Monitor glucose trend during exercise and increase fluid intake.

Glucose Level:	Hypoglycaemia or Hyperglycaemia - Not safe to start exercise
DO NOT EXERCISE – If hypoglycaemic event within the previous 24 h that required assistance from another individual to treat the event.<br>DO NOT EXERCISE – If hypoglycaemic event within the previous 24 h that did not require assistance but the intended exercise is potentially unsafe (e.g. swimming, skiing, surfing, etc.).<br>DO NOT EXERCISE UNTIL SYMPTOMS IMPROVE – If feeling unwell (e.g. abnormal sweating, trembling, anxiety, hunger, weakness, dizziness, inability to think straight).	
**<4.0mmol/L**	Delay Exercise – Treat hypoglycaemia: Consume one serve of fast acting carbohydrates and re-test after 15 min. If still wishing to exercise, ensure glucose level is ≥5.5mmol/L and follow up with one serve of slow acting carbohydrate. Do low to moderate intensity exercise and closely monitor glucose, re-test every 15 min.<br>DO NOT EXERCISE – If alone or type of exercise is potentially unsafe (e.g. swimming, skiing, surfing etc.).<br>**If glucose level is frequently <4.0mmol/L, schedule review with a Diabetes Healthcare Professional.**
**>15.0mmol/L**	DO NOT EXERCISE – If feeling unwell, tired, weak, thirsty and/or frequently urinating.<br>**If glucose level is frequently >15.0mmol/L, schedule review with a Diabetes Healthcare Professional.**

Fast Acting Carbohydrate (15g=one serve) examples: One serve as initial treatment	Slow Acting Carbohydrate (15g=one serve) examples: One serve as follow up treatment
- 100mL Lucozade<br>- 3 teaspoons honey, jam or sugar<br>- 3 glucose tablets<br>- 7 small or 4 large jelly beans<br>- 150mL fruit juice or soft drink<br>- 30mL cordial (non diet) mixed with 150mL water	- 250mL plain milk<br>- 2 sweet plain biscuits<br>- I tub (200g) yoghurt<br>- 1 piece of fruit<br>- 1 slice of bread<br>- next meal (if served within 30 min)

Guidelines for During Exercise

- Trained individuals have greater reductions in glucose during aerobic exercise than individuals with reduced cardiorespiratory fitness.
- High-intensity exercise or resistance exercise before aerobic exercise will attenuate the decrease in glucose compared to aerobic exercise alone.
- Completing an aerobic exercise cool down after high-intensity or resistance exercise will attenuate the glucose rise compared to performing high-intensity or resistance exercise alone.

**Glucose Level:**	**Below target glucose - Exercise with caution**
**<5.5mmol/L**	- Consume fast acting carbohydrates if next meal not planned within 30 min – one serve per hour with gentle exercise, two serves per hour with moderate-intensity exercise, four serves per hour with vigorous or high-intensity exercise. Alternative approach = 0.3-0.5g carbohydrate per kg of body mass per hour activity.<br>**If this occurs frequently, schedule review with Diabetes Healthcare Professional.**
**Glucose Level:**	**Rising glucose - Exercise with caution**
**Rises above pre-exercise level**	- Ensure medications have not been missed.<br>- Rise is more likely with higher intensity exercise such as weight lifting, sprints and racing.<br>- Rise may also be due to food consumed within the last 90 min.<br>- Monitor the rise but be prepared for the fall in glucose later – may require correction in carbohydrate consumption.
**Glucose Level:**	**Hypoglycaemia - Not safe to continue exercise**
**<4.0mmol/L**	STOP EXERCISING – Consume one serve of fast acting carbohydrate and re-check after 15 min. If glucose is still <4.0mmol/L repeat one serve fast acting carbohydrate. Once glucose is ≥4.0mmol/L consume one serve slow acting carbohydrate if next meal is more than 30 min away.<br>- Only resume exercise when glucose is ≥5.5mmol/L.<br>**- If this occurs frequently schedule review with a Diabetes Healthcare Professional.**

If the glucose level is of concern and/or is within orange or red areas of the Action Plan recurrently, the following should be discussed and reviewed with a Diabetes Healthcare Professional.

- Type of medications to lower glucose
- Timing of medications
- Glucose trend prior to exercise
- Timing and amount of previous food intake
- Presence and severity of diabetes complications
- Use of other medications secondary to diabetes
- Intensity, duration and type of exercise
- Time of day conducting exercise
