# Supplementary material for: Resources to Guide Exercise Specialists Managing Adults with Diabetes
Source: Sports Med Open. 2019 Jun 3;5:20. doi: 10.1186/s40798-019-0192-1 (PMC6546780; doi:10.1186/s40798-019-0192-1)

Type 1 Diabetes

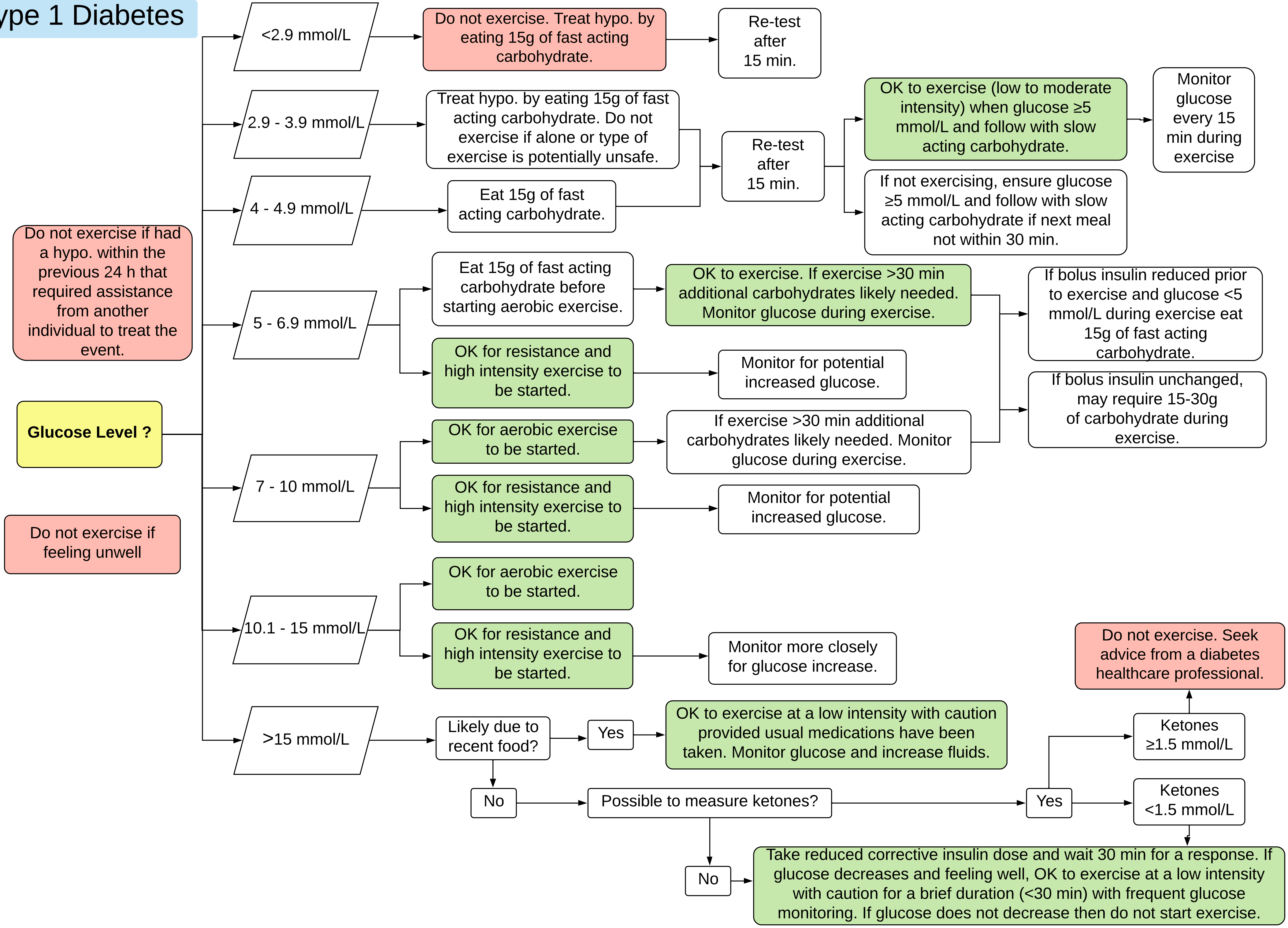

Supplement: Supplementary file 4 — Type 1 Diabetes. (PDF 49 kb) [file 40798_2019_192_MOESM4_ESM.pdf]
