# Supplementary figures and images for: Resources to Guide Exercise Specialists Managing Adults with Diabetes
Source: Sports Med Open. 2019 Jun 3;5:20. doi: 10.1186/s40798-019-0192-1 (PMC6546780; doi:10.1186/s40798-019-0192-1)

# Type 2 Diabetes

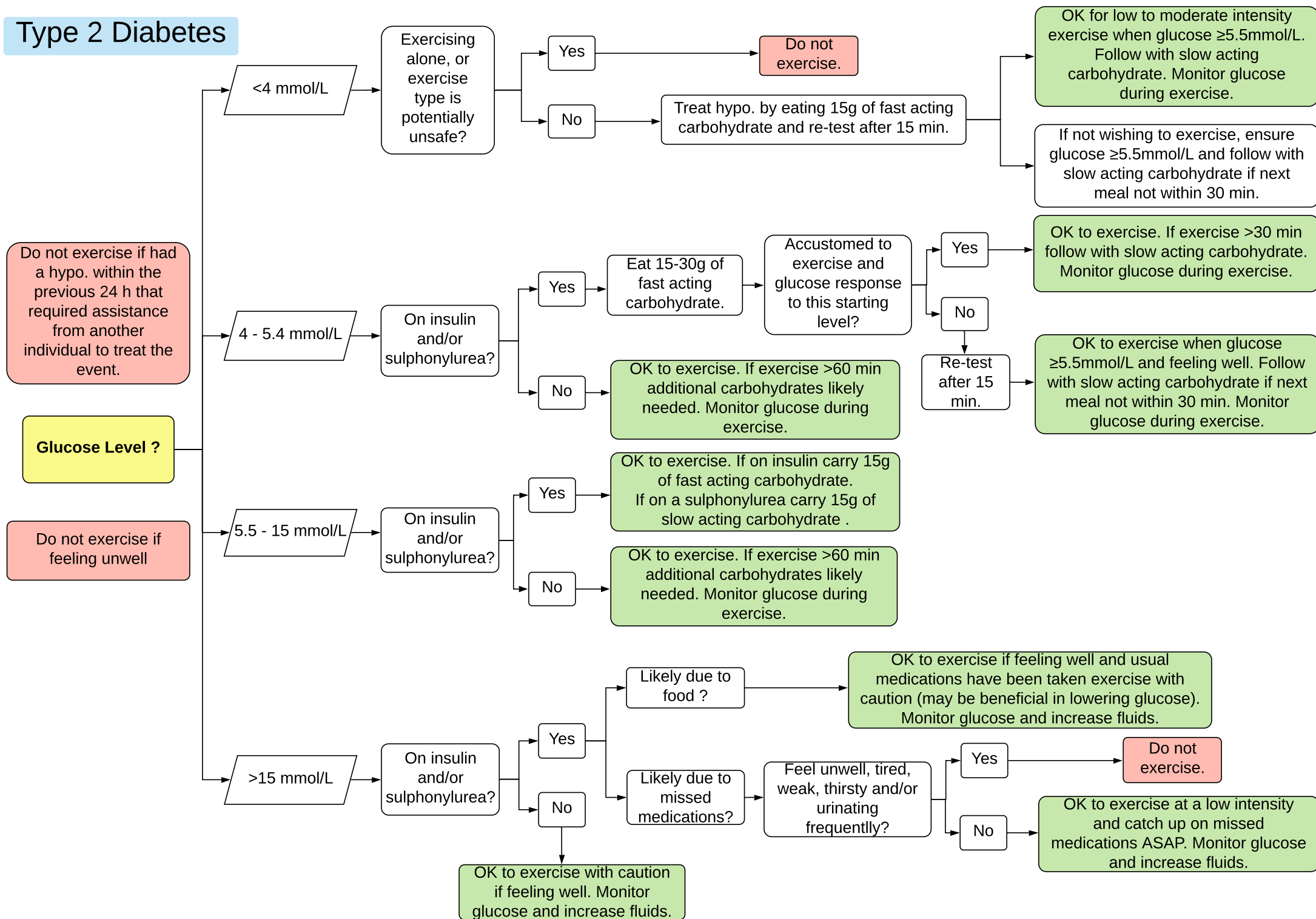

Supplement: Supplementary file 5 — Type 2 Diabetes. (PDF 48 kb) [file 40798_2019_192_MOESM5_ESM.pdf]
