# Supplementary material for: Resources to Guide Exercise Specialists Managing Adults with Diabetes
Source: Sports Med Open. 2019 Jun 3;5:20. doi: 10.1186/s40798-019-0192-1 (PMC6546780; doi:10.1186/s40798-019-0192-1)
Supplement: Supplementary file 6 — Additional Considerations for People with Diabetes Exercising. (PDF 78 kb) [file 40798_2019_192_MOESM6_ESM.pdf]

Table 1: Additional Considerations for People with Diabetes Exercising
Every person with diabetes is different, tailor the exercise plan to meet individual needs.
Assess the presence and severity of diabetes complications.
If previous foot or nerve problems check feet for blisters and ulcers before and after exercise.
Individuals with foot ulcers should avoid weight-bearing exercise that puts pressure on foot wounds.
When beginning or modifying an exercise program, monitor glucose for several hours before and after exercise to observe the trend.
Use of continuous glucose monitoring (via a transcutaneous sensor) provides greater detail regarding glucose changes, allowing finely-tuned medication adjustment.
Hypoglycaemia is the main risk for people with diabetes that exercise. It may lead to a loss of consciousness and a diabetic coma, that is life threatening.
For a person with type 1 diabetes about to exercise at a high intensity a small correction insulin dose is recommended if glucose is > 6.9 mmol/L. This dose can be then taken away from next meal bolus.
People with diabetes should consider exercising with a partner to assist in the detection of hypoglycaemia.
Be aware of the timing of medication administration; in particular, be aware of insulin action profiles (e.g. for short/rapid acting vs long acting insulins).
Consider effects of other medications: e.g. diuretics – fluid balance.<br>e.g. beta-blockers – attenuate heart rate response to exercise; may mask hypoglycaemia symptoms of palpitations/racing heart.<br>e.g. sodium-glucose co-transporter-2 (SGLT2) inhibitors – may cause severe acidosis with relatively normal glucose levels [41]. If feeling unwell after starting an SGLT2 inhibitor, postpone exercise and seek medical review.
A person with type 1 diabetes taking an SGLT-inhibitor must be able to check ketones due to risk of ketosis including euglycaemic ketosis [41].
Awareness of the 15 min delay between a blood glucose reading and a continuous glucose monitor's (CGM's) interstitial reading is important when planning exercise, especially when glucose level is low (e.g. a hypoglycaemic event has been treated and the blood glucose level is 5.5 mmol/L but the CGM may be measuring 4.5 mmol/L due to the delay).
Diabetes may lead to cardiac autonomic dysfunction and a blunted heart rate and blood pressure response to exercise. Therefore, additional monitoring of blood pressure and the use of a rating of perceived exertion (RPE) to monitor exercise intensity may be needed.
Insulin sensitivity varies diurnally, therefore different glucose responses may be observed with the same exercise undertaken at different times of the day.
One of the safest times to exercise with the lowest variation in glucose response to exercise (i.e. easier to predict) is in the morning before breakfast (dependent on glucose level).
A person with a glucose level frequently within the red area of the Action Plan should be reviewed by a Diabetes Healthcare Professional.
Individuals with retinopathy should avoid higher intensity aerobic and resistance exercises (with large increases in systolic blood pressure), head-down activities, jumping or jarring activities. These all increase haemorrhage risk.
Appropriate fluid intake is necessary to minimise dehydration and risk of heat stress. Increasing fluid intake is important when the glucose level is high.
